# Activation of the DDR Pathway Leads to the Down-Regulation of the TGFβ Pathway and a Better Response to ICIs in Patients With Metastatic Urothelial Carcinoma

**DOI:** 10.3389/fimmu.2021.634741

**Published:** 2021-06-18

**Authors:** Chaozheng Zhou, Anqi Lin, Manming Cao, Weimin Ding, Weiming Mou, Ningyi Guo, Zhenyu Chen, Jian Zhang, Peng Luo

**Affiliations:** ^1^ The Department of Oncology, Zhujiang Hospital, Southern Medical University, Guangzhou, China; ^2^ The First Clinical Medical School, Southern Medical University, Guangzhou, China

**Keywords:** DDR pathway, immunotherapy, immune microenvironment, immune checkpoint inhibitors, urothelial cancer, TGFβ pathways

## Abstract

Immune checkpoint inhibitors (ICIs) have changed the treatment paradigm of metastatic urothelial carcinoma (mUC), a dominant type of bladder cancer (BC). Previous studies have shown an association between gene mutations in the DNA damage response (DDR) pathway and the immunotherapy response in mUC but have neglected the effect of the activation level of the DDR pathway on the ICI response in mUC. A published immunotherapy cohort with genome, transcriptome and survival data for 348 mUC patients was used. An external cohort (The Cancer Genome Atlas Bladder Cancer) and the GSE78220 cohort were used for validation. The activation level of the DDR pathway was quantified using single-sample gene set enrichment analysis (ssGSEA). Further analysis on the genome, immunogenicity, and the immune microenvironment was conducted using the DDR ssGSEA enrichment score-high (DSSH) group and the DDR ssGSEA enrichment score-low (DSSL) group. In the mUC cohorts, the DSSH group was associated with longer overall survival times (P=0.026; Hazard ratio=0.67; 95%CI: 0.46−0.95). The DSSH group was also associated with higher tumor mutation burden, neoantigen load, immune-activated cell patterns, and immune-related gene expression levels. The GSEA results indicated an immune activation state in DSSH group, which correlated with a down-regulation in the transforming growth factor β receptor signaling pathway. Our study suggests that the activation level of the DDR pathway may be a novel predictive marker for immunotherapy efficacy in patients with mUC.

## Introduction

Bladder cancer (BC) originates from the urinary tract system and is the 10th most common tumor worldwide; its incidence in men is significantly higher than that in women (1). In addition, 80% of BCs are urothelial carcinoma (UC), which originates from transitional epithelial cells (2). The first-line treatment for patients with malignant UC is still platinum-based chemotherapy (3), but nearly half of patients experience cancer progression or recurrence after treatment (4). Notably, due to the extremely high tumor mutation burden (TMB) in UC, the immunogenicity of UC is also very high (5). This fact indicates that immunotherapy could be efficacious in patients with UC. In 2016, the US Food and Drug Administration (FDA) approved 5 immune checkpoint inhibitors (ICIs) for the treatment of cisplatin-resistant advanced UC (3). Among them, atezolizumab and pembrolizumab are approved as first-line treatments for cisplatin-resistant mUC (the other three are nivolumab, durvalumab and avelumab). Immune checkpoint-blocking (ICB) therapy has shown good and long-lasting results in some UC patients (6). However, only 20-30% of UC patients fully respond to ICI therapy (7). In addition, ICI therapy can cause adverse events (AEs) and immune-related adverse events (irAEs) in some patients (8). In the absence of accurate and effective biomarkers, it is difficult for doctors to predict the efficacy of ICI therapy in UC patients and patient prognosis. Therefore, to improve the response rate of UC patients to ICI therapy as well as patient prognosis, a biomarker that can predict the efficacy of ICI therapy is urgently needed.

In previous studies, a series of corresponding biomarkers, including TMB, tumor neoantigen load (NAL), PD-L1 expression level, microsatellite instability (MSI), tumor microenvironment (TME) markers and T cell gene expression profiles (GEPs) (9, 10), have been found to predict the response of cancer to ICI therapy as well as patient prognosis. In addition, combining TMB with PD-L1 expression or other markers significantly improves the accuracy and effectiveness of the prediction results (11). From the tumor genome to the tumor immune microenvironment, each of the markers above reflects certain characteristics of the tumor, which are closely related to each other. Therefore, more exploratory work is needed to identify the factors that regulate most of the existing biomarkers.

The DNA damage response (DDR) pathway is a monitoring mechanism in the cell that is activated and plays a corresponding role when DNA damage occurs to maintain the integrity and stability of the cell genome (12). Generally, the DDR pathway contains eight sub-pathways that can be divided into two main mechanisms. The pathways initiated in response to single-stranded DNA damage include base excision repair (BER), nucleotide excision repair (NER), mismatch repair (MMR), and single-stranded DNA binding (SSB), while the pathways activated during double-stranded DNA damage include nonhomologous end joining (NHEJ), homologous recombination (HR), the Fanconi anemia (FA) pathway and double-strand break repair (DSB) (13-15). All of the sub-pathways above are connected to each other to form a network that induces the DDR pathway in response to DNA damage.

The DDR pathway plays an important role in the occurrence and evolution of UC and is closely related to the clinical prognosis of UC patients. Teo et al. found that in patients with advanced BC treated with cisplatin, gene mutations in the DDR pathway are associated with a favorable clinical prognosis (16). In recent years, with the development and application of immunotherapy for tumor treatment, the importance of the DDR pathway has been highlighted. Yuanquan Yang et al. noted that MSH4 gene mutations in the MMR pathway are closely related to a complete response in patients with metastatic BC after ICI treatment (17). Teo et al. found that other DDR pathway alterations can also predict the efficacy of ICI therapy in tumor treatment (18). These results indicate a strong link between the DDR pathway and ICI therapy response. However, few studies have focused on the activation level of the DDR pathway, and how it influences immunotherapy in UC patients still requires further exploration.

In view of the information above, we mainly analyzed how the activation level of the DDR pathway affects the efficacy of ICI therapy in UC patients from the aspects of the tumor genome, tumor immunogenicity, and the tumor immune microenvironment. According to the results of this study, a high activation level of the DDR pathway indicates a favorable prognosis and immune response in UC patients receiving ICI treatment and may serve as a biomarker for predicting immunotherapy response.

## Materials and Methods

### The Relationship Between the Activation Level of the DDR Pathway and the Prognosis of Immunotherapy-Treated Urothelial Carcinoma Patients

Data licensed under Creative Commons 3.0 from http://research-pub.gene.com/IMvigor210CoreBiologies/ were downloaded *via* the IMvigor210CoreBiologies package (7); the data included the genome, transcriptome and corresponding clinical characteristics of 348 patients with metastatic urothelial carcinoma (mUC) who received atezolizumab (an anti-PD-L1 agent) treatment. The same type of data was obtained from The Cancer Genome Atlas Bladder Cancer (TCGA-BLCA) cohort *via* the University of California, Santa Cruz (UCSC) Xena database (19). In this study, only samples with prognostic information were retained. Next, we collected gene sets related to the DDR pathway and 8 sub-pathways from the Molecular Signatures Database (MSigDB) (20) (**Supplementary Table 1**). We defined the DDR pathway as the sum of the remaining eight sub-pathways. After converting the RNA-seq count matrix to Transcripts Per Million (TPM) (21) matrix, we used the GSVA package (22) to perform single-sample gene set enrichment analysis (ssGSEA). Based on the enrichment scores (ESs) of samples in each cohort in each pathway, we performed Kaplan-Meier (KM) survival analysis and compared the number of responders. We used the ssGSEA ESs to reflect the activation level of the pathways in the samples, and the median was utilized to determine the activation level for further grouping. A cohort of patients with metastatic melanoma treated with pembrolizumab or nivolumab (PD-1 blocker) was used to verify the predictive effect of the DDR pathway activation level on ICI efficacy. The RNA-seq data and clinical data of this cohort were obtained from the (n=27) Gene Expression Omnibus (GEO) GSE78220 dataset (23).

### Analysis of Genome Mutation Characteristics and Tumor Immunogenicity

To clarify the effect of the DDR pathway on UC mutation characteristics and immunogenicity, we compared the difference between the DDR ssGSEA ES-high (DSSH) group and the DDR ssGSEA ES-low (DSSL) group in terms of TMB and NAL. We also performed the above comparison in the sub-pathway-related groups. TMB was calculated according to the practice of Chalmers et al. (24), which defines the number of nonsynonymous mutations as the original mutation count and divides this value by 38 Mb to quantify TMB; this calculation was applied in the mUC cohort and TCGA BC cohort. To visualize the gene mutation rate of DDR-related genes and the clinical characteristics in each cohort, we used the ComplexHeatmap package (25). Genomic information in the TCGA-BLCA cohort was included in our comparative analysis as well (26).

### Analysis of Immune Characteristics in Urothelial Carcinoma

To better understand the relationship between the DDR pathway and **immune characteristics** of UC, we first used the DESeq2 package to perform differential gene analysis between the DSSH and DSSL groups in the TCGA-BLCA cohort (27). Genes with adjusted p<0.05 and |log2 foldchange(FC)|>0.5, were recognized as differentially expressed genes (DEGs). We compared the two groups to analyze differences in the expression of immune-related genes and their functional classifications, as reported by Vesteinn Thorsson et al. (26). The CIBERSORT method (28) was considered to quantify the relative abundance of immune cells in the samples from each cohort and to compare these abundance between the DSSH and DSSL groups. In addition, immune-related information from the TCGA-BLCA cohort was compared between the DSSH and DSSL groups (26).

### Gene Set Enrichment Analysis (GSEA)

We used the gene analysis results produced by the DESeq2 package as the input and sorted the genes according to their logFC values. We performed GSEA with the ClusterProfiler package (29). Finally, gene ontology (GO), Kyoto Encyclopedia of Genes and Genomes (KEGG) pathway and Reactome pathway enrichment analysis were performed, and only the terms with p values less than 0.05 were considered to be significantly enriched.

### Internal Association Between the DDR Pathway and the TGFβ Signaling Pathway

We downloaded the drug sensitivity data and gene expression profile data of BC cell lines from the Genomics of Drug Sensitivity in Cancer (GDSC) database (30). The gene expression profile matrix was standardized, and the same method was used to calculate the ES of each cell line and to group the cell lines. Three inhibitors of the transforming growth factor β (TGFβ) signaling pathway were selected for drug sensitivity analysis. The gene set of the TGFβ signaling pathway from MSigDB was collected to quantify the activation level by ssGSEA.

### Data Analysis

For comparisons of factors such as overall survival (OS), TMB, NAL, immune cell fraction and abundance between the DSSH and DSSL groups, we used the Mann-Whitney U test. Fisher’s exact test and Chi-square test were used to analyze the contingency table. We utilized Spearman’s correlation coefficient for the correlation analysis. The KM method and the log-rank test were applied in the survival analysis. When performing the survival analysis, the survminer package was used to calculate the cutoff of each cohort according to the relationship between the survival result and the ES of each pathways with the maximally selected rank statistics (31). The median value was selected to distinguish the level of pathway activation and compare the differences in the number of responders among different groups. The univariate Cox proportional hazard regression model was established to calculate the hazard ratio. The multivariate Cox regression model was used to determine independent prognostic factors. In this study, p<0.05 was considered significant, and all statistical tests were two-tailed. In addition, we used the ggpubr package (32) to create box plots. All statistical analyses and visualization work were performed using R software (https://www.r-project.org/; version 4.0.0).

## Results

### Activation of the DDR Pathway Improves the Therapeutic Effect of ICIs on Patients With mUC

**Figure 1:**
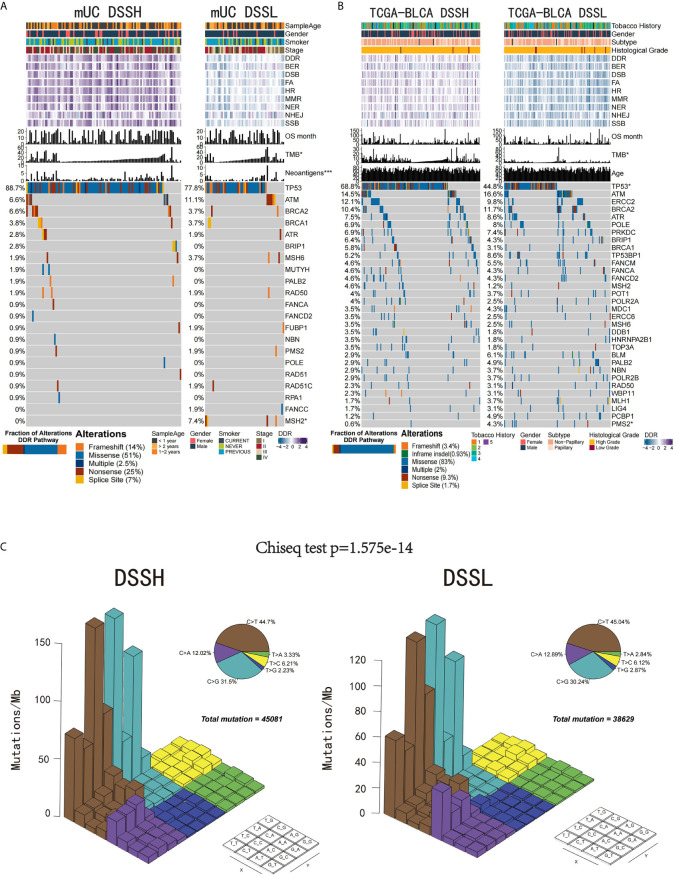
The relationship between the activation level of the DDR pathway and genomic and clinical features. **(A)** Gene mutations in the DDR pathway in the mUC cohort. The samples are sorted according to TMB, and the genes are sorted according to mutation frequency (left side). Patient age, sex, smoking level, tumor stage, DDR-related pathway activation level, OS time, TMB and number of tumor neoantigens are annotated at the top. TP53 and MSH2 gene mutation frequencies were significantly different between the two groups. The bar plot in the bottom left corner shows the proportion of different alterations in the DDR pathway. **(B)** The top 32 genes with the highest frequency of mutations in DDR-related pathways in the TCGA-BLCA cohort. Smoking history, sex, tumor subtype, race, DDR-related pathway activation level, OS time, TMB and age are annotated at the top. The PMS2 gene mutation frequency was significantly different between the two groups. The bar plot in the bottom left corner shows the proportion of different alterations in the DDR pathway. **(C)** The Lego diagram shows the difference in mutation patterns between the DSSH (left) and DSSL groups (right). The pie chart in the upper right corner shows the proportion of samples with the six mutation patterns. The data were analyzed by the chi-square test, and a difference in mutation patterns was found between the DSSH and DSSL groups (p=1.6e-14). *p < 0.05; ***p < 0.001.

The flowchart of our work is presented in **Supplementary Figure 1**. We first studied whether the activation level of the DDR pathway affects specific genomic and clinical features of UC patients. We used the ssGSEA method to quantitatively analyze the activation level of the DDR pathway of samples in the mUC cohort and TCGA-BLCA cohort and divided the samples into DSSH and DSSL groups according to the median value. The results showed that there was no significant difference in clinical characteristics between the DSSH and DSSL groups in either cohort. In the mUC cohort, both TMB and NAL were significantly higher in the DSSH group than in the DSSL group (TMB, p=0.043; NAL, p=0.00029). In the TCGA-BLCA cohort, TMB was also significantly higher in the DSSH group (p=0.019). In terms of the mutation frequency of DDR-related genes, the TP53 gene had a higher mutation rate in the DSSH group than in the DSSL group (mUC cohort, p=0.099; TCGA cohort, p=9.9e-06). In addition, most of the gene mutations in DDR-related pathways were missense mutations, followed by nonsense mutations, in both cohorts (**Figures 1A, B**). Notably, the mutations in the DSSH and DSSL groups of the TCGA-BLCA cohort were mainly C>G and C>T mutations (**Figure 1C**), and there were significant differences in the mutation rates between the groups (Chi-squared test, p=1.6 e-14).

**Figure 2:**
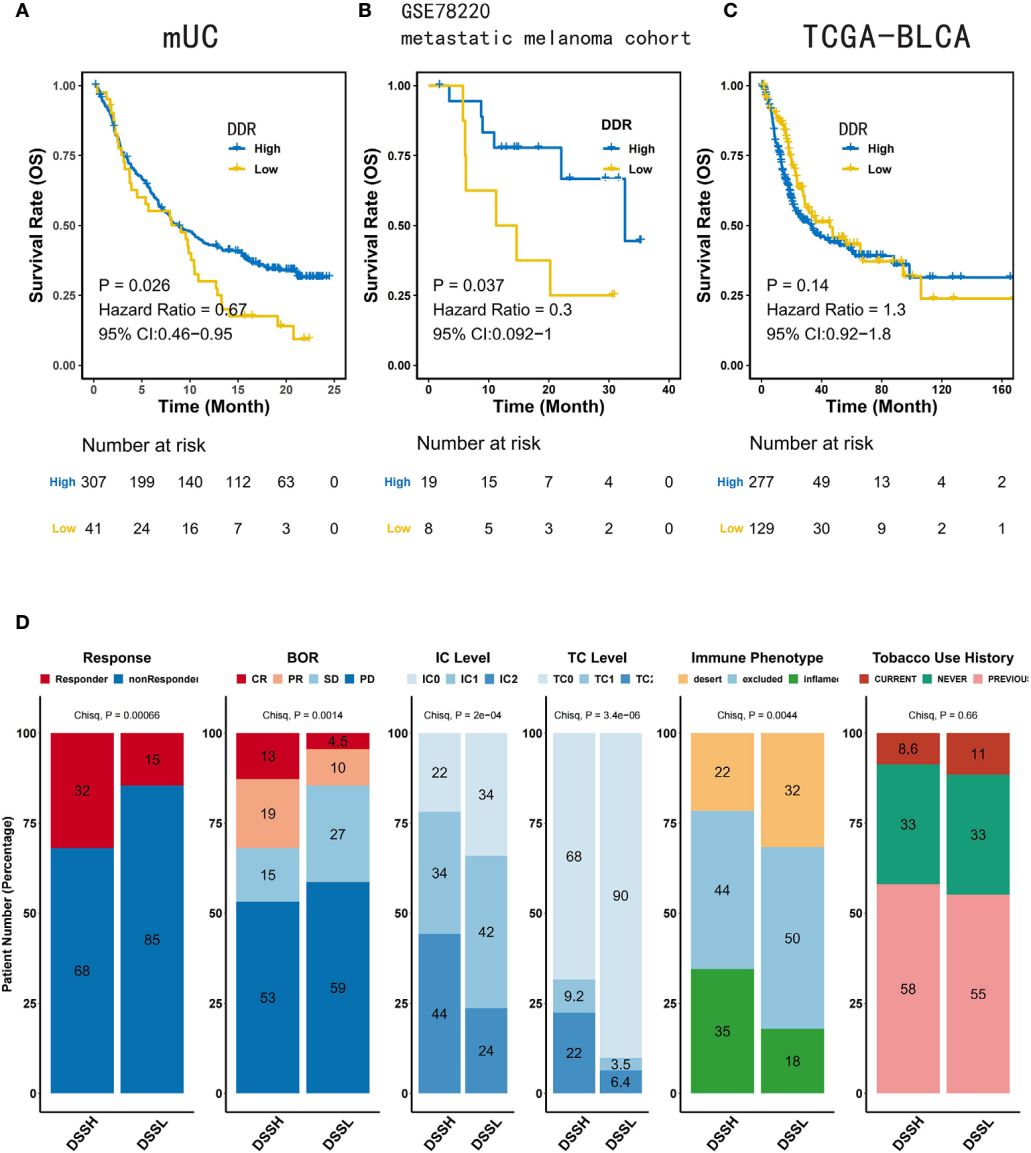
Activation of the DDR pathway indicates a better prognosis and treatment response in mUC patients treated with ICIs. **(A)** KM survival analysis of the relationship between the activation status of the DDR pathway and OS time of mUC patients treated with ICIs. **(B)** KM survival analysis of the relationship between the activation status of the DDR pathway and OS time of metastatic melanoma patients treated with ICIs. **(C)** KM survival analysis of the relationship between the activation status of the DDR pathway and OS time of TCGA-BLCA cohort. **(D)** The number of responders and the distribution of clinicopathological parameters in DSSH and DSSL groups. The DSSH group positively correlated with more responders, better response and higher PD-L1 expression (IC/TC), but negatively correlated with immune excluded phenotype. The distribution of tobacco use history was uniform between the two groups.

To evaluate whether the activation level of the DDR pathway can predict the prognosis of mUC patients and the efficacy of ICI treatment, we first grouped the mUC cohorts according to the ES of the DDR-related pathways and performed KM survival analysis. mUC patients with a high level of the DDR pathway activation showed a significantly longer OS time than those with a low activation level (Hazard ratio=0.67, [95% CI, 0.46-0.95], p=0.026) (**Figure 2A**). We used the same method for survival analysis and found that the activation level of DDR pathway significantly affected the prognosis of ICI-treated metastatic melanoma patients (Hazard ratio=0.3, [95% CI, 0.092-1] (**Figure 2B**) but not in the TCGA-BLCA cohort (**Figure 2C**). Similar results were obtained in the study of other DDR-related pathways in three cohorts (**Supplementary Figures 2** and **4A**). Multivariate Cox regression indicated the activation level of the DDR pathway (p=0.024) along with the immune phenotype (p=0.006) as independent prognostic factors (**Supplementary Figure 3B**).

According to the response to treatment, mUC patients were divided into responder (complete or partial response, CR or PR) and non-responder (stable or progressive disease, SD or PD) groups. In the analysis of clinicopathological characteristics, there were more ICI responders, and better best overall response (BOR) in the DSSH group. Higher PD-L1 expression in tumor cells (TC) or immune cells (IC), and more inflamed phenotypes were also allocated in the DSSH group. These characteristics indicate that patients in DSSH group may show better anti-tumor immune response (**Figure 2D**). Besides, the activation of other DDR-related pathways can also improve patient response to ICI (**Supplementary Figure 3**). In the metastatic melanoma cohort, the number of responders to ICI was not affected by the DDR pathway (**Supplementary Figure 4B**).

### Activation of the DDR Pathway Enhances Tumor Immunogenicity and Promotes Antitumor Immunity

**Figure 3:**
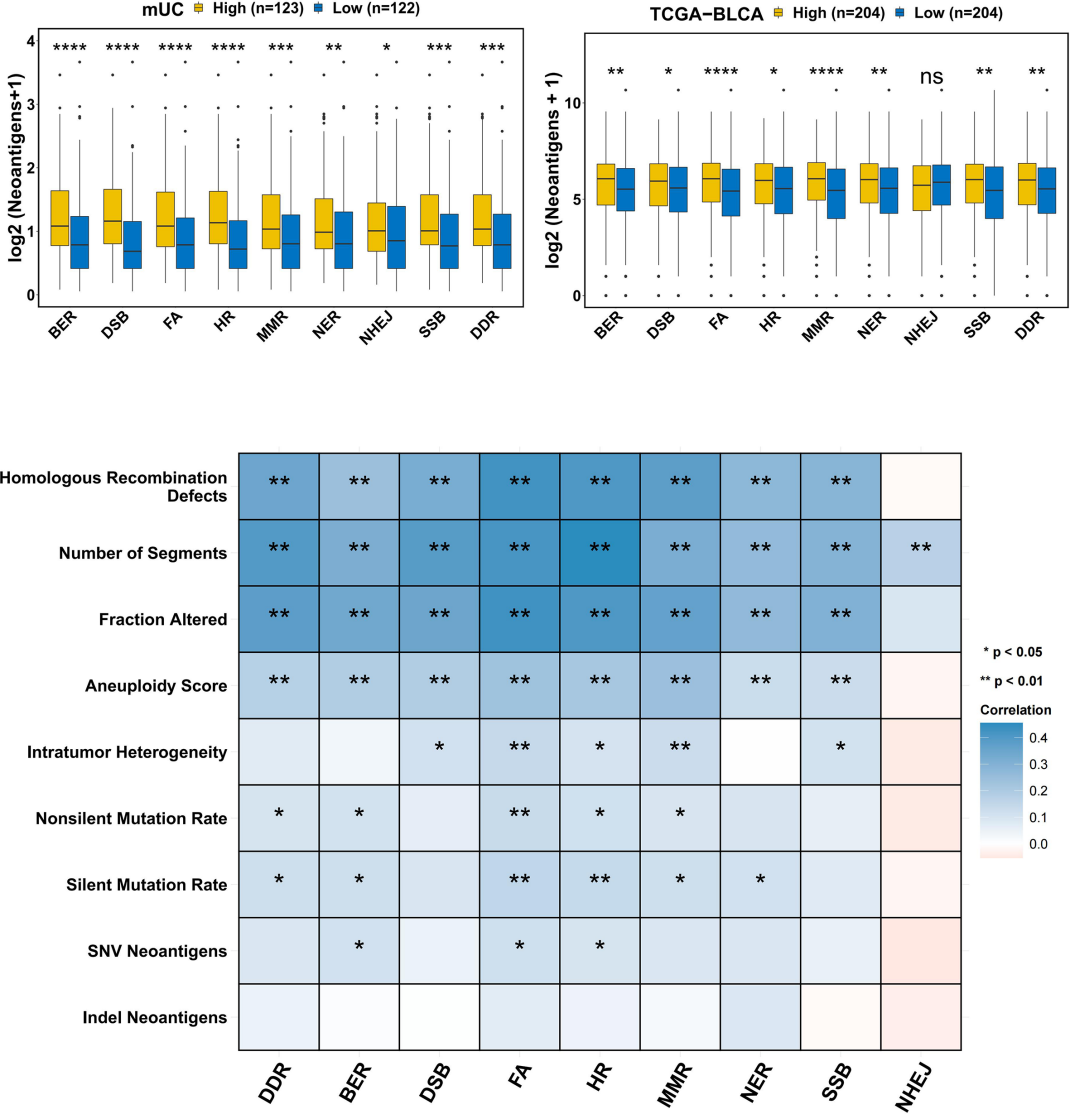
The relationship between the DDR pathway and tumor immunogenicity. **(A)** Comparison of the tumor neoantigens between groups with different levels of DDR-related pathway activation in the mUC cohort. A higher activation level of DDR-related pathways was accompanied by a significant increase in tumor neoantigens. **(B)** Comparison of tumor neoantigens between groups with different activation levels in DDR-related pathways in the TCGA-BLCA cohort. There were significant differences in the NAL according to the activation level of the DDR-related pathways except for NHEJ. **(C)** Correlation matrix of DDR-related pathways and indel neoantigens, SNV neoantigens, silent mutation rate, nonsilent mutation rate, intratumor heterogeneity, number of segments, fraction altered, aneuploidy score and HR defect score. Blue indicates a positive correlation, while red indicates a negative correlation. *p < 0.05; **p < 0.01; ***p < 0.001; ****p < 0.0001.

In order to determine the effect of the DDR pathway and related pathways on tumor immunogenicity, we compared the differences in the NAL of patients with different activation levels of the DDR pathway and related pathways in the mUC cohort. It is worth noting that patients with DDR pathway activation in the mUC cohort showed significantly higher NAL levels than those without DDR activation (p=0.00027) (**Figure 3A**), and similar results were found in the TCGA-BLCA cohort (p=0.0025) (**Figure 3B**). We found no significant difference between the DSSH and DSSL groups in TCR repertoire analysis, but in cancer testis antigen(CTA) overall expression(p=0.031) (**Supplementary Figure 5**). In the process of studying the correlation between the DDR pathway and various tumor genome features in the TCGA-BLCA cohort, we observed that the number of segments, the fraction altered, the aneuploidy score, and the HR defect score were significantly positively correlated with the activation level of DDR-related pathways (**Figure 3C**).

**Figure 4:**
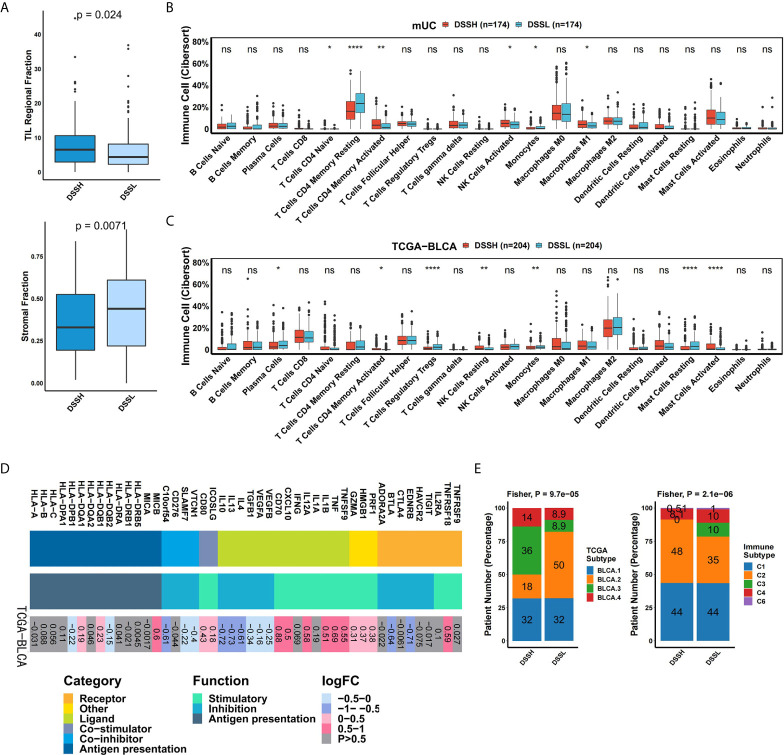
Activation of the DDR pathway affects the immune microenvironment of mUC. **(A)** The results of hematoxylin and eosin (H&E)-stained slices from the TCGA-BLCA cohort. The box plot shows the difference in stromal fraction and TIL fraction between the DSSH and DSSL groups. **(B)** The box plot shows the difference in 22 immune cells between the DSSH and DSSL groups according to the CIBERSORT analysis results of the mUC cohort. **(C)** The box plot shows the difference in 22 immune cell types between the DSSH and DSSL groups according to the CIBERSORT analysis results of the TCGA-BLCA cohort. **(D)** The difference in the average expression of immune-related genes between the DSSH and DSSL groups in the TCGA-BLCA cohort is shown in the heat maps. From left to right are the gene name, gene type and function, and the direction of change in gene expression. Genes of the same category or function are represented by the same color. Red in the right rectangle indicates upregulation, blue indicates downregulation, and gray indicates that the result is not significant (p>0.05). The logFC value is marked on the right rectangle. **(E)** The proportion of samples of different TCGA subtypes and immune subtypes in the DSSH group and DSSL groups in the TCGA-BLCA cohort. Fisher’s exact test was used for data analysis. There was a significant difference between the DSSH and DSSL groups in terms of subtype composition (TCGA subtype, p=9.7e-5; Immune subtype, p=2.1e-6) TCGA molecular subtype is based on the RNA-seq data of 129cases of urothelial bladder carcinoma and has different transcriptome and genomic characteristics (33). Six immune subtypes refer to C1 (wound healing), C2 (IFN-g dominant), C3 (inflammatory), C4 (lymphocyte depleted), C5 (immunologically quiet), C6 (TGF-b dominant) (26). *p < 0.05; **p < 0.01; ***p < 0.001; ****p < 0.0001.

The TME plays an important regulatory role in the evolution of BC. We hypothesized that the DDR pathway might promote ICI treatment by affecting the TME. First, analysis of the TME indicated that the DSSH group had a significantly higher tumor-infiltrating lymphocyte (TIL) fraction (p=0.024) and a lower stromal fraction (p=0.0071) than the DSSL group (**Figure 4A**). According to the CIBERSORT algorithm, we calculated the relative enrichment of 22 immune cell types in each patient. In the DSSH group of the mUC cohort, the proportion of activated memory CD4 T cells (p=0.0021), activated natural killer (NK) cells (p=0.017), and M1 macrophages (p=0.044) was significantly higher than that in the DSSL group, while the proportion of monocytes (p=0.011) was significantly lower (**Figure 4B**). In the DSSH group of the TCGA-BLCA cohort, the proportion of activated memory CD4 T cells (p=0.032), resting NK cells (p=0.0032), and activated mast cells (p=2.3e-5) was significantly higher than that in the DSSL group, while the proportion of Treg cells (p=7.8e-6) and monocytes (p=3.6e-3) was significantly lower (**Figure 4C**). In addition, genes with immune-activation functions, such as TNF, IL1, and CXCL10 (logFC>0.5, adjusted p<0.05), were significantly upregulated in the DSSH group, while genes with immunosuppressive functions, such as IL4, IL13, and IL10, were significantly downregulated in the DSSH group (logFC< -0.5, adjusted p<0.05) (**Figure 4D**). In terms of the TCGA subtypes, the DSSH group had more BLCA2-type and fewer BLCA3-type (‘basal/squamous-like’) patients than the DSSL group (Fisher’s exact test: p=9.7e-5) (**Figure 4E**). In terms of the immune subtypes, there were more type C2 (IFN-g dominant) patients in the DSSH group than in the DSSL group, while the C3 (inflammatory) patients were mostly found in the DSSL group (Fisher’s exact test: p=2.1e-6) (**Figure 4E**). The results above suggested that a high level of DDR pathway activation may improve tumor immunogenicity and enhance antitumor activity in mUC.

### Activation of the DDR Pathway in mUC Is Negatively Associated With the TGFβ Pathway

**Figure 5:**
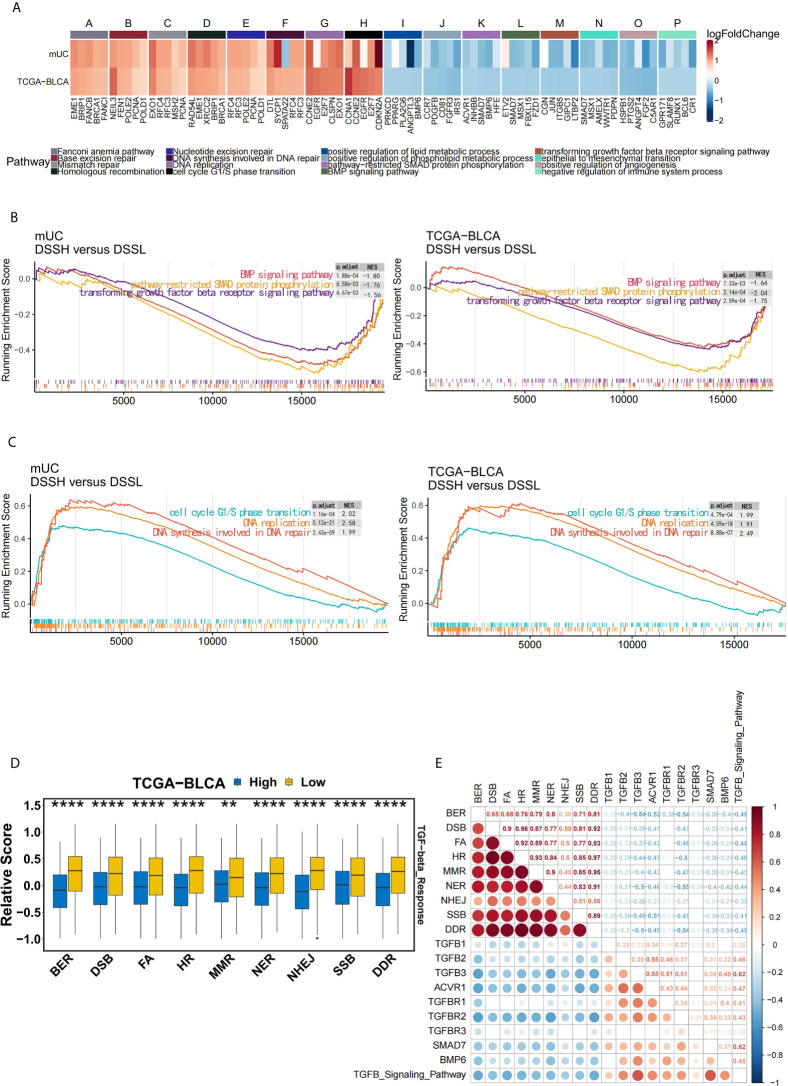
The activation of DDR pathway is accompanied by the down-regulation of the TGFβ pathway in mUC cohort. **(A)** The heat map shows the changes in the average expression of the top 5 core genes in the partially significantly enriched pathways in the DSSH group of both cohorts. Red indicates upregulation, while blue indicates downregulation. The expression levels of core genes in DDR-related pathways, cell cycle and DNA synthesis-related pathways were upregulated in the DSSH group, while the expression levels of core genes in the TGFβ signaling pathway and lipid metabolism-related pathways were downregulated in the DSSH group (adjusted p value < 0.05). **(B)** Cell cycle G1/S transition- and DNA synthesis-related pathways were significantly enriched in the DSSH group. **(C)** The BMP signaling pathway, SMAD protein phosphorylation, and the TGFβ receptor signaling pathway were significantly enriched in the DSSH group. **(D)** The relationship between DDR-related pathways and the TGFβ response pathway in the TCGA-BLCA cohort. The relevant data of the TCGA-BLCA cohort were derived from a study by Vesteinn Thorsson et al. (26). **(E)** The relationship between the degree of activation of DDR related pathway and the degree of activation of TGFB pathway and the expression of its core genes. The size and color of the circle reflect the degree of correlation between the two. Red indicates a positive correlation, while blue indicates a negative correlation. The number represents the Spearman correlation coefficient. *p < 0.05; **p < 0.01; ***p < 0.001; ****p < 0.0001.

We further explored the possible mechanism by which mUC patients with high activation of DDR pathways benefit from ICI therapy. **Figure 5A** shows the differential expression profiles of core genes in DDR-related pathways, TGFβ-related signaling pathways and cell proliferation pathways. The transforming growth factor superfamily contains multiple members, including TGFβ and bone morphogenetic proteins (BMPs). The results of GSEA showed that TGFβ-related signaling pathways was significantly down-regulated in the DSSH group (**Figure 5B**), while cell proliferation-related pathways were significantly up-regulated (**Figure 5C**).

Remarkably, in the TCGA-BLCA cohort, patients with suppression of DDR pathways exhibited a significant increase in the level of the TGF-β response (p=1.8e-8) (**Figure 5D**). At the same time, in the mUC cohort, there was a significant negative correlation between the activation of DDR pathway, activation of the TGFβ signaling pathway and the expression of related core genes (**Figure 5E**). However, the half-maximal inhibitory concentration (IC50) values of the three TGFβ signaling pathway inhibitors in BC cell lines did not differ significantly due to the activation of DDR pathway (**Supplementary Figure 6**).

## Discussion

Although the discovery of ICIs has changed the treatment of mUC patients, only a small number of patients completely or partially respond to ICIs and benefit from them. Moreover, immunotherapy use is accompanied by a risk of irAEs. Therefore, to achieve better antitumor immune responses from ICI treatment and obtain better prognostic results, predictive markers are urgently required. In this study, through survival analysis, we found that in the mUC cohort treated with ICIs, a high activation level of the DDR pathway can improve the outcome of ICI treatment and the prognosis of mUC patients. This result indicated that the activation level of the DDR pathway can function as a biomarker for ICI treatment in mUC patients to some extent. In the TCGA-BLCA samples, the information was insufficient to classify patients according to treatment. It may be that the activation of the DDR pathway can predict the prognosis of mUC samples treated with atezolizumab but has no predictive effect on the prognosis of primary tumor samples in the TCGA-BLCA cohort receiving conventional chemotherapy and radiotherapy. Notably, the DSSH group had more TP53 mutations than the DSSL group; Andrea Necchi et al. noted that TP53 mutations are associated with higher levels of leukocyte infiltration at the pancancer level (26), suggesting that the immune response might be stronger in the DSSH group. Follow-up studies indicated that the DDR pathway may affect the efficacy of ICIs by affecting tumor immunogenicity and the tumor immune microenvironment.

mUC with activation of the DDR pathway was characterized by higher immunogenicity. This feature was mainly manifested as a significant increase in TMB and NAL in mUC. In addition, a positive correlation between the activation level of the DDR pathway and the number of segments, the fraction altered, the aneuploidy score, and the HR defect score indicated the instability of the tumor genome. The GSEA results also showed that cell cycle-related pathways and DNA synthesis pathways, especially those that occur during DNA repair, were significantly enriched in the DSSH group. Therefore, a higher activation level of the DDR pathway was highly associated with a higher mutation burden. Further studies showed that activation of the DDR pathway induces an antitumor immune microenvironment. In the DSSH group, which has a higher TIL fraction, some immune-activating genes were upregulated, while some immunosuppressive genes were downregulated. Moreover, a high activation level of the DDR pathway was accompanied by a significant increase in the abundance of activated CD4 cells and a significant decrease in the abundance of Treg cells. Based on previous studies, CD4+ T cells are related to the production of effective antitumor immune responses, while Treg cells inhibit the antitumor immune microenvironment by secreting cytokines IL10 and TGFβ in the tumor microenvironment (34). Therefore, the abundance of activated CD4 T cells may increase in DSSH tumors to facilitate the secretion of more immune-activating factors and reduce the abundance of Treg cells to suppress the release of immunosuppressive factors, thereby promoting the antitumor activities and improving the efficacy of ICI treatment.

Additionally, our study demonstrated that the DSSH group had a significantly reduced response to TGFβ compared with that in the DSSL group and also had downregulated expression of TGFB1. Through GSEA of the two cohorts, we found that the activation level of the TGFβ receptor signaling pathway and other related pathways was significantly downregulated in the DSSH group. The TGFβ signaling pathway plays a regulatory role in many biological processes, including cell growth, cell differentiation, apoptosis, metastasis, and cancer evolution (35). Although the activation of TGFβ and its signaling pathways plays an antitumor role in the early stages of cancer, these pathways promote EMT and angiogenesis and mediate immunosuppression to promote tumor progression and metastasis. Moreover, many malignant tumors produce excessive amounts of TGFβ (36), thus promoting malignancy. It is worth noting that there is a significant negative correlation between the activation of DDR pathway and the activation of the TGFβ signaling pathway and the expression of core genes such as TGFB3, ACVR1 and TGFBR2 in mUC. In terms of drug sensitivity, we did not find that the sensitivity of BC cell lines to three different TGFβ pathway inhibitors changed significantly due to the activation of DDR pathway. This finding may be due to the lack of tumor immune microenvironment of tumor tissue in BC cell line. Constance J. Martin’s paper notes that the selective inhibition of TGFB1 can change the tumor immune landscape and improve tumor tissue resistance to ICI therapy (37). Sanjeev Mariathasan found that fibroblasts in mUC attenuated tumor response to PD-L1 blockers through the TGFβ response (7). Based on the above pathway analysis and correlation analysis, we speculate that the activation of DDR pathway may inhibit the expression of the TGFβ signaling pathway through the tumor immune microenvironment. However, further functional verification is needed.

Collectively, our study demonstrates the specific value of DDR pathway in predicting the efficacy of ICI in patients with mUC from the perspective of transcriptome. Compared with previous studies, which predict the efficacy of ICI from mutations in the DDR pathway (38), our study provides a new perspective. In clinical practice, although DDR pathway mutations are relatively easy to detect, most patients can only accept targeted gene sequencing of hundreds of genes using ctDNA. Additionally, our previous study found that targeted gene sequencing is not as accurate as whole genome sequencing (WES) (39). Considering that the impact of different mutation types or mutation site classification of DDR mutations on prediction is not very clear, predicting the therapeutic effect of ICI from the overall activation level of DDR pathway has unique advantages.

Although we successfully noted that the activation level of the DDR pathway may be an effective predictor of ICI treatment response in mUC patients, our research still has some limitations. First, we have not validated our findings in mUC patients who have received immunotherapy. Second, the results of functional enrichment analysis need further *in vivo* and *in vitro* validation. Because cohorts of mUC patients that have received immunotherapy and have available follow-up data, gene expression data, and genome data are rather rare, it was difficult for us to verify our findings in mUC patients who have received ICI treatment in our hospital. Therefore, we used the metastatic melanoma cohort to observe whether DDR ES can predict the outcome of ICI treatment in other cancers. We hope to perform corresponding cell experiments or animal experiments in the future to determine the influence of the activation level of the DDR pathway on the immune microenvironment of mUC and to clarify the detailed mechanism underlying the efficacy of ICI treatment. Finally, this study did not find a uniform cutoff value, but used the median of the ES of the DDR pathway and DDR related pathways to group patients; we hope to find a more consistent cutoff in future research.

## Conclusions

Our study provided evidence that the activation level of the DDR pathway is associated with longer OS times and with known immunotherapy response markers, including the TMB, NAL, immune-related genes, and the abundance of specific immune cells. In addition, the TGFβ pathway is in a state of low expression when DDR pathway is activated. All of these factors contribute to a better response to ICIs in mUC. Therefore, the state of the DDR pathway could be a predictive biomarker for ICIs. A series of prospective clinical studies and molecular mechanistic explorations are needed to support our findings.

## Data Availability Statement

The original contributions presented in the study are included in the article/**Supplementary Material**. Further inquiries can be directed to the corresponding authors.

## Author Contributions

Conceptualization: JZ and PL. Formal analysis: CZ, AL, and MC. Resources: CZ, JZ, and PL. Software: CZ, AL, WD, and MC. Supervision: JZ and PL. Visualization: CZ, AL, and WD. Writing–original draft: CZ, AL, and MC. Writing–review and editing: CZ, AL, WD, WM, NG, ZC and MC. All authors contributed to the article and approved the submitted version.

## Conflict of Interest

The authors declare that the research was conducted in the absence of any commercial or financial relationships that could be construed as a potential conflict of interest.

## Supplementary Material

Supplementary Figure 1 Brief flowchart of this study.

Supplementary Figure 2 The relationship between the level of activation of DDR sub-pathway and the prognosis of patients in mUC cohort and TCGA-BLCA cohort. **(A)** KM survival analysis was used to assess the relationship between the activation level of DDR-related pathways and the OS time of patients in the mUC cohort. **(B)** KM survival analysis was used to assess the relationship between the activation level of DDR-related pathways and the OS time of patients in the TCGA-BLCA cohort.

Supplementary Figure 3 Activation of the DDR pathway shows specific prognostic value for ICI-treated mUC. **(A)** The high activation of eight DDR sub-pathways was accompanied by more responders to ICI. Chi squared test was used for data analysis. **(B)** Multivariate Cox regression model shows the hazard ratio and 95% confidence interval (95% CI) of the DDR pathway and other clinical parameter or prior reported biomarkers. Higher activation level of the DDR pathway is a protective factor collaborated with the inflamed immune phenotype (DDR: p = 0.024; Immune phenotype, p = 0.006). *p < 0.05; **p < 0.01; ***p < 0.001.

Supplementary Figure 4 Activation of the DDR pathway indicates a favorable prognosis in patients with metastatic melanoma treated with ICIs. **(A)** KM survival analysis was used to assess the relationship between the activation status of DDR-related pathways and the OS time of patients with metastatic melanoma treated with ICIs. **(B)** The ratio of responders in groups with high or low activation levels of DDR-related pathways. Fisher’s exact test was used for data analysis.

Supplementary Figure 5 Differential analysis between the DSSH and DSSL groups of the TCGA-BLCA cohort in TCR repertoire and cancer testis antigen overall expression (CTA).

Supplementary Figure 6 Drug sensitivity to three inhibitors of the TGFβ signaling pathway in BC cell lines was not affected by the activation of the DDR pathway. The target of A-83-01 and LY2109761 is TGFB1. The targets of SB505124 are TGFBR1, ACVR1B and ACVR1C.

Supplementary Table 1 List of genes included in DNA damage response (DDR) gene set used for Comparison Analysis (MsigDB).

## References

[1] Bray F Ferlay J Soerjomataram I Siegel RL Torre LA Jemal A. Global Cancer Statistics 2018: GLOBOCAN Estimates of Incidence and Mortality Worldwide for 36 Cancers in 185 Countries. CA Cancer J Clin (2018) 68(6):394–424. 10.3322/caac.21492 30207593

[2] Willis D Kamat AM. Nonurothelial Bladder Cancer and Rare Variant Histologies. Hematol Oncol Clin North Am (2015) 29(2):237–52. 10.1016/j.hoc.2014.10.011 25836932

[3] Patel VG Oh WK Galsky MD. Treatment of Muscle-Invasive and Advanced Bladder Cancer in 2020. CA Cancer J Clin (2020) 70(5):404–23. 10.3322/caac.21631 32767764

[4] Oing C Rink M Oechsle K Seidel C Von Amsberg G Bokemeyer C. Second Line Chemotherapy for Advanced and Metastatic Urothelial Carcinoma: Vinflunine and Beyond - A Comprehensive Review of the Current Literature. J Urol (2016) 195(2):254–63. 10.1016/j.juro.2015.06.115 26410730

[5] Necchi A Madison R Pal SK Ross JS Agarwal N Sonpavde G. Comprehensive Genomic Profiling of Upper-tract and Bladder Urothelial Carcinoma. Eur Urol Focus (2020). 10.1016/j.euf.2020.08.001 32861617

[6] Balar AV Galsky MD Rosenberg JE Powles T Petrylak DP Bellmunt J. Atezolizumab as First-Line Treatment in Cisplatin-Ineligible Patients With Locally Advanced and Metastatic Urothelial Carcinoma: A Single-Arm, Multicentre, Phase 2 Trial. Lancet (2017) 389(10064):67–76. 10.1016/S0140-6736(16)32455-2 27939400 PMC5568632

[7] Mariathasan S Turley SJ Nickles D Castiglioni A Yuen K Wang Y. Tgfβ Attenuates Tumour Response to PD-L1 Blockade by Contributing to Exclusion of T Cells. Nature (2018) 554(7693):544–8. 10.1038/nature25501 PMC6028240 29443960

[8] Postow MA Sidlow R Hellmann MD. Immune-Related Adverse Events Associated With Immune Checkpoint Blockade. N Engl J Med (2018) 378(2):158–68. 10.1056/nejmra1703481 29320654

[9] Bai R Lv Z Xu D Cui J. Predictive Biomarkers for Cancer Immunotherapy With Immune Checkpoint Inhibitors. Biomarker Res (2020) 8(1):34. 10.1186/s40364-020-00209-0 PMC7450548 32864131

[10] Cristescu R Mogg R Ayers M Albright A Murphy E Yearley J. Pan-Tumor Genomic Biomarkers for PD-1 Checkpoint Blockade-Based Immunotherapy. Science (2018) 362(6411):eaar3593. 10.1126/science.aar3593 30309915 PMC6718162

[11] Rizvi H Sanchez-Vega F La K Chatila W Jonsson P Halpenny D Darragh. Molecular Determinants of Response to Anti-Programmed Cell Death (PD)-1 and Anti-Programmed Death-Ligand 1 (PD-L1) Blockade in Patients With non-Small-Cell Lung Cancer Profiled With Targeted Next-Generation Sequencing. J Clin Oncol (2018) 36(7):633–41. 10.1200/JCO.2017.75.3384 PMC6075848 29337640

[12] Zhou BBS Elledge SJ. The DNA Damage Response: Putting Checkpoints in Perspective. Nature (2000) 408(6811):433–9. 10.1038/35044005 11100718

[13] Nickoloff JA Jones D Lee SH Williamson EA Hromas R. Drugging the Cancers Addicted to DNA Repair. J Natl Cancer Institute (2017) 109(11). 10.1093/jnci/djx059 PMC5436301 28521333

[14] Nastasi C Mannarino L D’incalci M. DNA Damage Response and Immune Defense. Int J Mol Sci (2020) 21(20):1–28. 10.3390/ijms21207504 PMC7588887 33053746

[15] Park S Lee H Lee B Lee SH Sun JM Park WY. Dna Damage Response and Repair Pathway Alteration and Its Association With Tumor Mutation Burden and Platinum-Based Chemotherapy in SCLC. J Thorac Oncol (2019) 14(9):1640–50. 10.1016/j.jtho.2019.05.014 31125737

[16] Teo MY Bambury RM Zabor EC Jordan E Al-Ahmadie H Boyd ME. DNA Damage Response and Repair Gene Alterations are Associated With Improved Survival in Patients With Platinum-Treated Advanced Urothelial Carcinoma. Clin Cancer Res (2017). 10.1158/1078-0432.CCR-16-2520 PMC5511570 28137924

[17] Yang Y Jain RK Glenn S Xu B Singh PK Wei L. Complete Response to anti-PD-L1 Antibody in a Metastatic Bladder Cancer Associated With Novel MSH4 Mutation and Microsatellite Instability. J Immunother Cancer (2020) 8(1):e000128. 10.1136/jitc-2019-000128 32221012 PMC7206971

[18] Teo MY Seier K Ostrovnaya I Regazzi AM Kania BE Moran MM. Alterations in DNA Damage Response and Repair Genes as Potential Marker of Clinical Benefit From PD-1/PD-L1 Blockade in Advanced Urothelial Cancers. J Clin Oncol (2018) 36(17):1685–94. 10.1200/JCO.2017.75.7740 PMC6366295 29489427

[19] Goldman MJ Craft B Hastie M Repecka K McDade F Kamath A. Visualizing and Interpreting Cancer Genomics Data Via the Xena Platform. Nat Biotechnol (2020) 38(6):675–8. 10.1038/s41587-020-0546-8 PMC7386072 32444850

[20] Subramanian A Tamayo P Mootha VK Mukherjee S Ebert BL Gillette M. Gene Set Enrichment Analysis: A Knowledge-Based Approach for Interpreting Genome-Wide Expression Profiles. Proc Natl Acad Sci U S A (2005). 10.1073/pnas.0506580102 PMC1239896 16199517

[21] Wagner GP Kin K Lynch VJ. Measurement of mRNA Abundance Using RNA-seq Data: RPKM Measure is Inconsistent Among Samples. Theory Biosci (2012) 131(4):281–5. 10.1007/s12064-012-0162-3 22872506

[22] Hänzelmann S Castelo R Guinney J. Gsva: Gene Set Variation Analysis for Microarray and RNA-Seq Data. BMC Bioinf (2013) 14(1):7. 10.1186/1471-2105-14-7 PMC3618321 23323831

[23] Hugo W Zaretsky JM Sun L Song C Moreno BH Hu-Lieskovan S. Genomic and Transcriptomic Features of Response to Anti-PD-1 Therapy in Metastatic Melanoma. Cell (2016) 165(1):35–44. 10.1016/j.cell.2016.02.065 26997480 PMC4808437

[24] Chalmers ZR Connelly CF Fabrizio D Gay L Ali DM Ennis R. Analysis of 100,000 Human Cancer Genomes Reveals the Landscape of Tumor Mutational Burden. Genome Med (2017) 9(1):34. 10.1186/s13073-017-0424-2 28420421 PMC5395719

[25] Gu Z Eils R Schlesner M. Complex Heatmaps Reveal Patterns and Correlations in Multidimensional Genomic Data. Bioinformatics (2016) 32(18):2847–9. 10.1093/bioinformatics/btw313 27207943

[26] Thorsson V Gibbs DL Brown SD Wolf D Bortone DS Ou Yang T-H. The Immune Landscape of Cancer. Immunity (2018) 48(4):812–30. 10.1016/j.immuni.2018.03.023 PMC5982584 29628290

[27] Love MI Huber W Anders S. Moderated Estimation of Fold Change and Dispersion for RNA-seq Data With Deseq2. Genome Biol (2014) 15(12):550. 10.1186/s13059-014-0550-8 25516281 PMC4302049

[28] Newman AM Liu CL Green MR Gentles AJ Feng E Xu Y. Robust Enumeration of Cell Subsets From Tissue Expression Profiles. Nat Methods (2015) 12(5):453–7. 10.1038/nmeth.3337 PMC4739640 25822800

[29] Yu G Wang LG Han Y He QY. ClusterProfiler: An R Package for Comparing Biological Themes Among Gene Clusters. Omi A J Integr Biol (2012) 16(5):284–7. 10.1089/omi.2011.0118 PMC3339379 22455463

[30] Yang W Soares J Greninger P Edelman EJ Lightfoot H Forbes S. Genomics of Drug Sensitivity in Cancer (Gdsc): A Resource for Therapeutic Biomarker Discovery in Cancer Cells. Nucleic Acids Res (2013) 41(D1):D955–61. 10.1093/nar/gks1111 PMC3531057 23180760

[31] Alboukadel K Marcin K Przemyslaw B Scheipl F. “Drawing Survival Curves Using ‘Ggplot2’ [R Package Survminer Version 0.4.3],” R Packag. Version 0.4.3. CRAN (2018).

[32] Kassambara A. Ggpubr’: ‘Ggplot2’ Based Publication Ready Plots,” R Packag. Version 0.2.5. CRAN (2020).

[33] Weinstein JN Akbani R Broom BM Wang W Verhaak RGW McConkey D. Comprehensive Molecular Characterization of Urothelial Bladder Carcinoma. Nature (2014) 507(7492):315. 10.1038/nature12965 24476821 PMC3962515

[34] Tay RE Richardson EK Toh HC. Revisiting the Role of CD4+ T Cells in Cancer Immunotherapy—New Insights Into Old Paradigms. Cancer Gene Ther (2020) 28(1–2):5–17. 10.1038/s41417-020-0183-x 32457487 PMC7886651

[35] Haque S Morris JC. Transforming Growth Factor-β: A Therapeutic Target for Cancer. Hum Vaccines Immunotherapeutics (2017) 13(8):1741–50. 10.1080/21645515.2017.1327107 PMC5557219 28575585

[36] Connolly EC Freimuth J Akhurst RJ. Complexities of TGF-β Targeted Cancer Therapy. Int J Biol Sci (2012) 8(7):964–78. 10.7150/ijbs.4564 PMC3399319 22811618

[37] Martin CJ Datta A Littlefield C Kalra A Chapron C Wawersik S. Selective Inhibition of Tgfβ1 Activation Overcomes Primary Resistance to Checkpoint Blockade Therapy by Altering Tumor Immune Landscape. Sci Transl Med (2020) 12:536. 10.1126/scitranslmed.aay8456 32213632

[38] Wang Z Zhao J Wang G Zhang F Zhang Z Zhang F. Comutations in DNA Damage Response Pathways Serve as Potential Biomarkers for Immune Checkpoint Blockade. Cancer Res (2018) 78(22):6486–96. 10.1158/0008-5472.CAN-18-1814 30171052

[39] Luo P Lin A Li K Wei T Zhang J. Ddr Pathway Alteration, Tumor Mutation Burden, and Cisplatin Sensitivity in Small Cell Lung Cancer: Difference Detected by Whole Exome and Targeted Gene Sequencing. J Thorac Oncol (2019) 14(12):e276–9. 10.1016/j.jtho.2019.08.2509 31757380

