# Supplementary figures and images for: Activation of the DDR Pathway Leads to the Down-Regulation of the TGFβ Pathway and a Better Response to ICIs in Patients With Metastatic Urothelial Carcinoma
Source: Front Immunol. 2021 Jun 18;12:634741. doi: 10.3389/fimmu.2021.634741 (PMC8253049; doi:10.3389/fimmu.2021.634741)

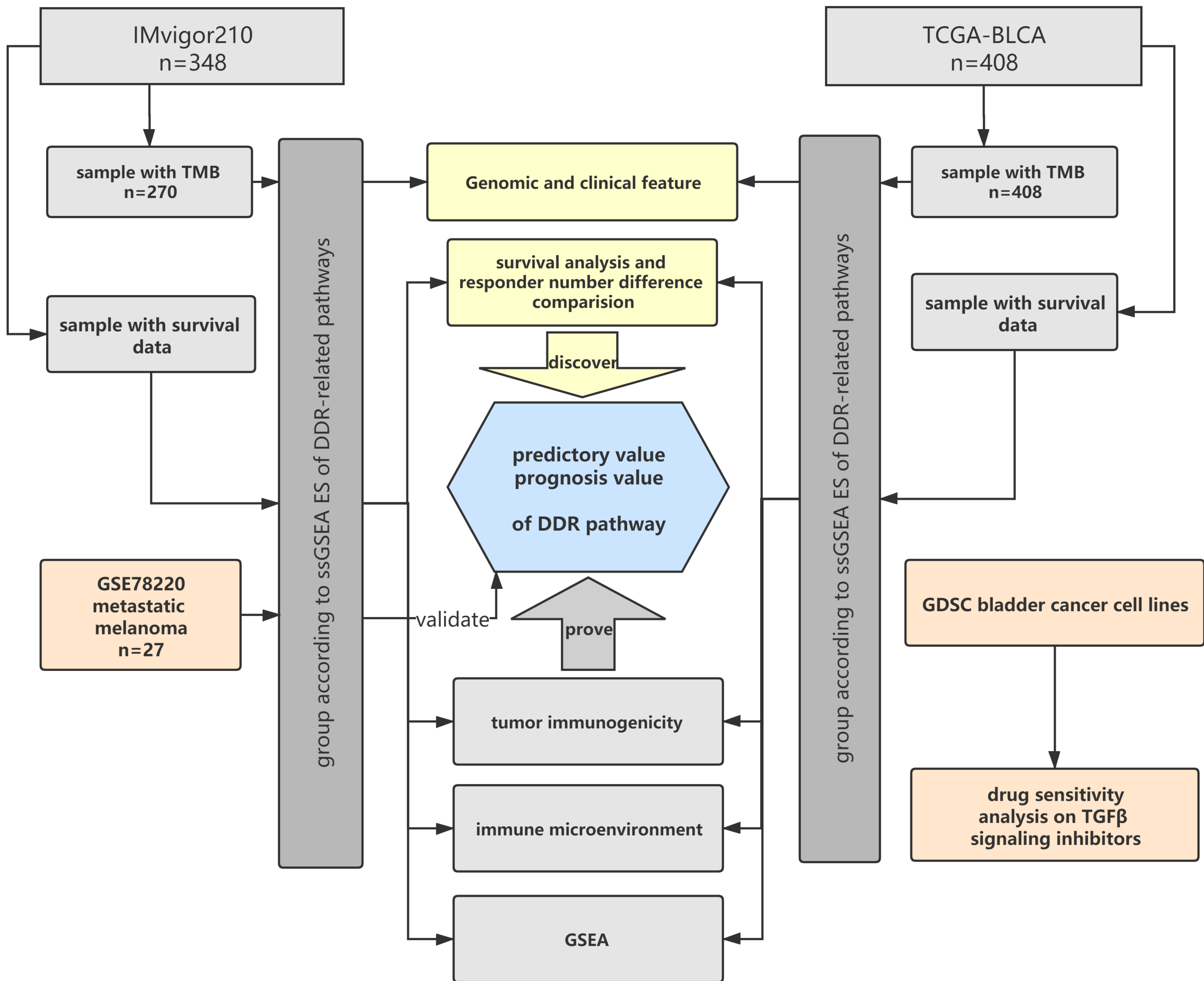

Supplement: Supplementary Figure 1 — Brief flowchart of this study. [file Image_1.pdf]

A

## mUC Cohort

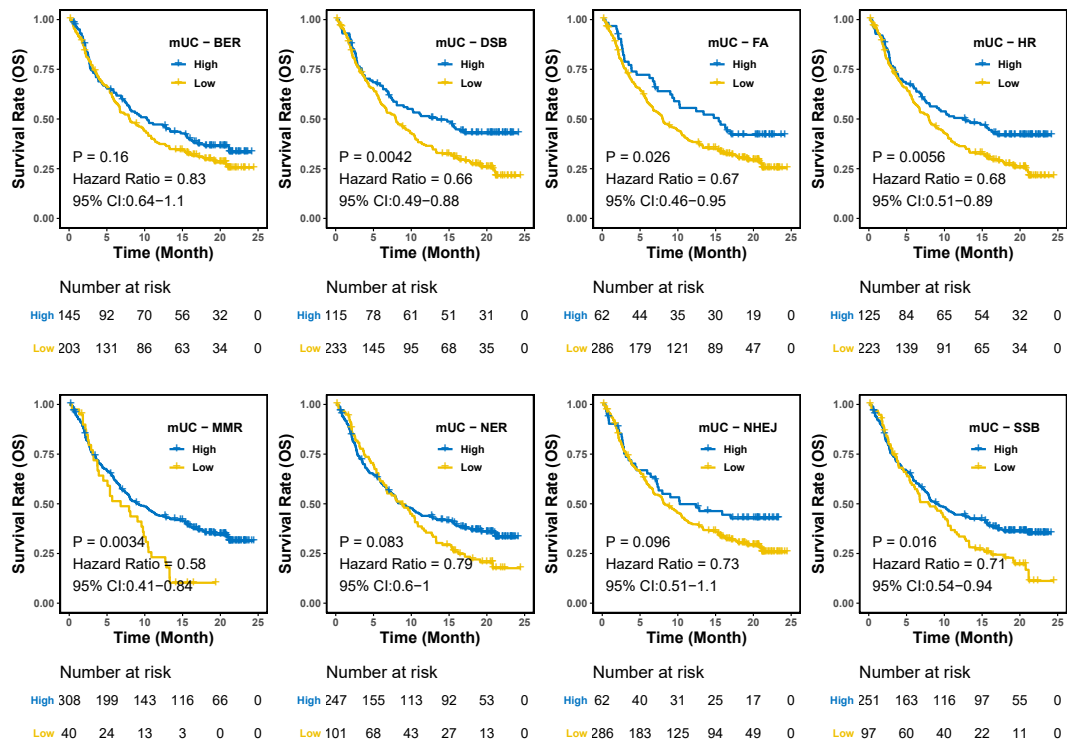

B

## TCGA-BLCA Cohort

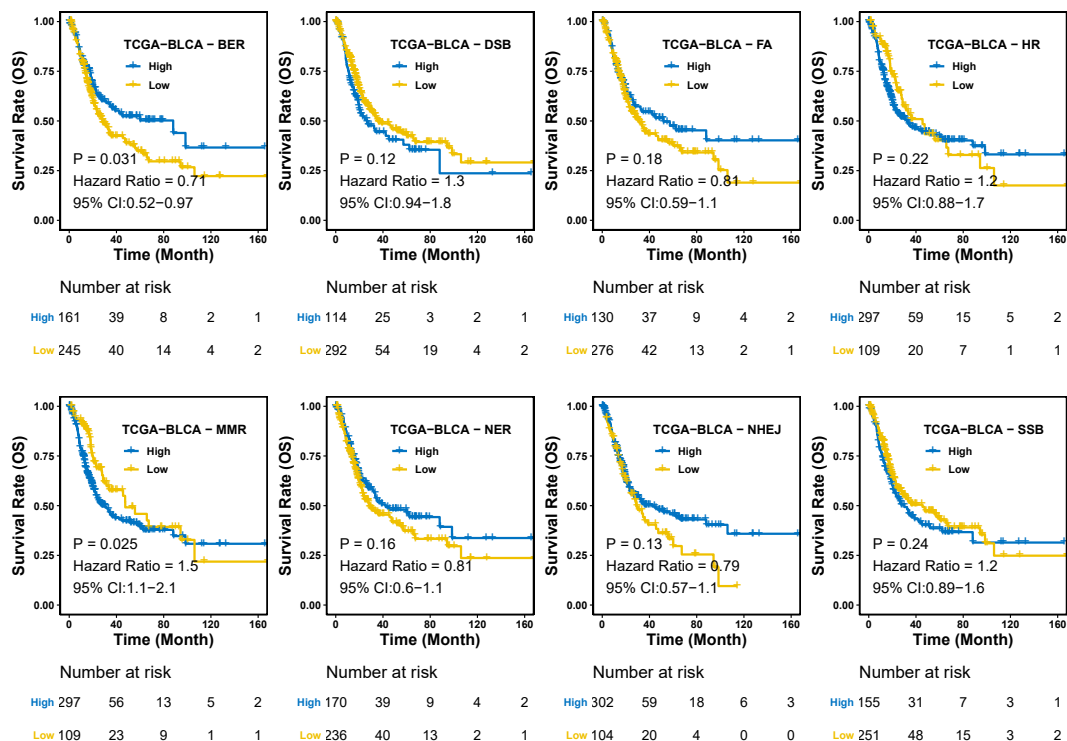

Supplement: Supplementary Figure 2 — The relationship between the level of activation of DDR sub-pathway and the prognosis of patients in mUC cohort and TCGA-BLCA cohort. (A) KM survival analysis was used to assess the relationship between the activation level of DDR-related pathways and the OS time of patients in the mUC cohort. (B) KM survival analysis was used to assess the relationship between the activation level of DDR-related pathways and the OS time of patients in the TCGA-BLCA cohort. [file Image_2.pdf]

A

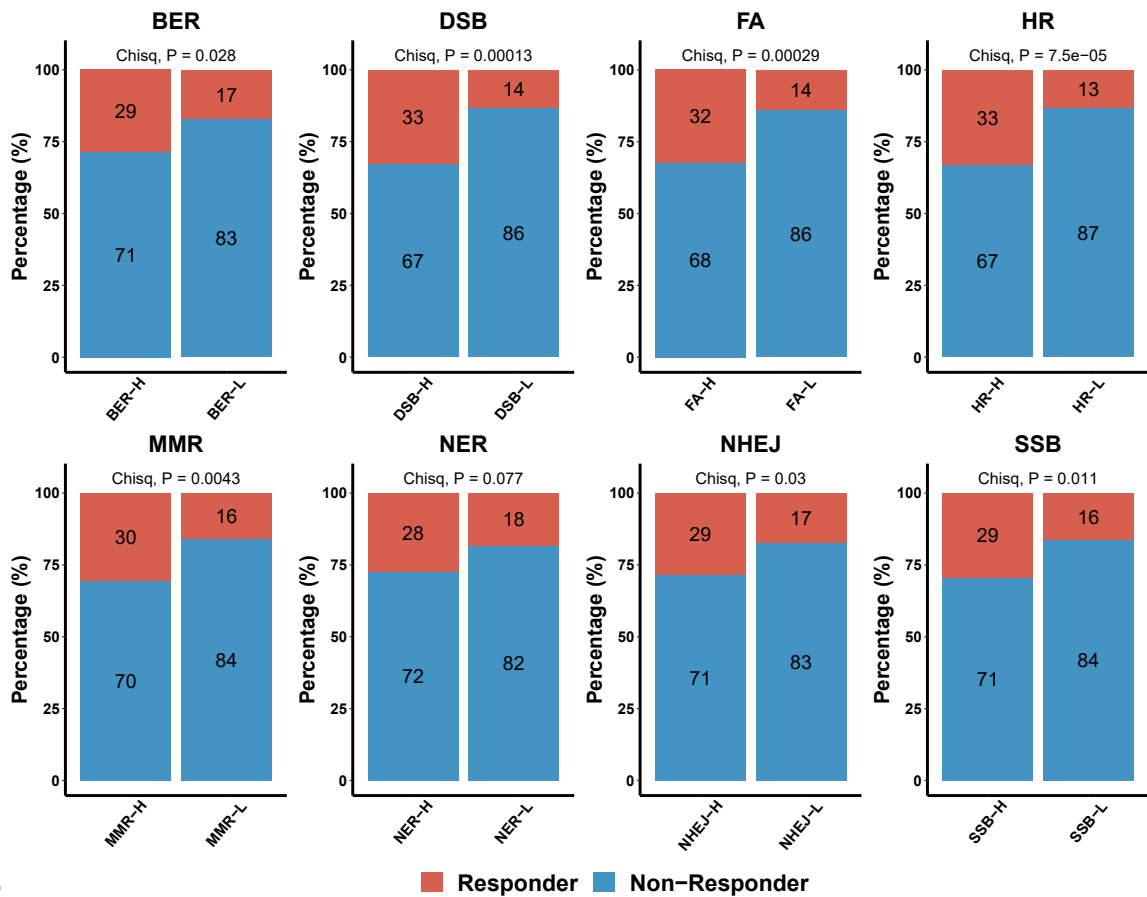

B

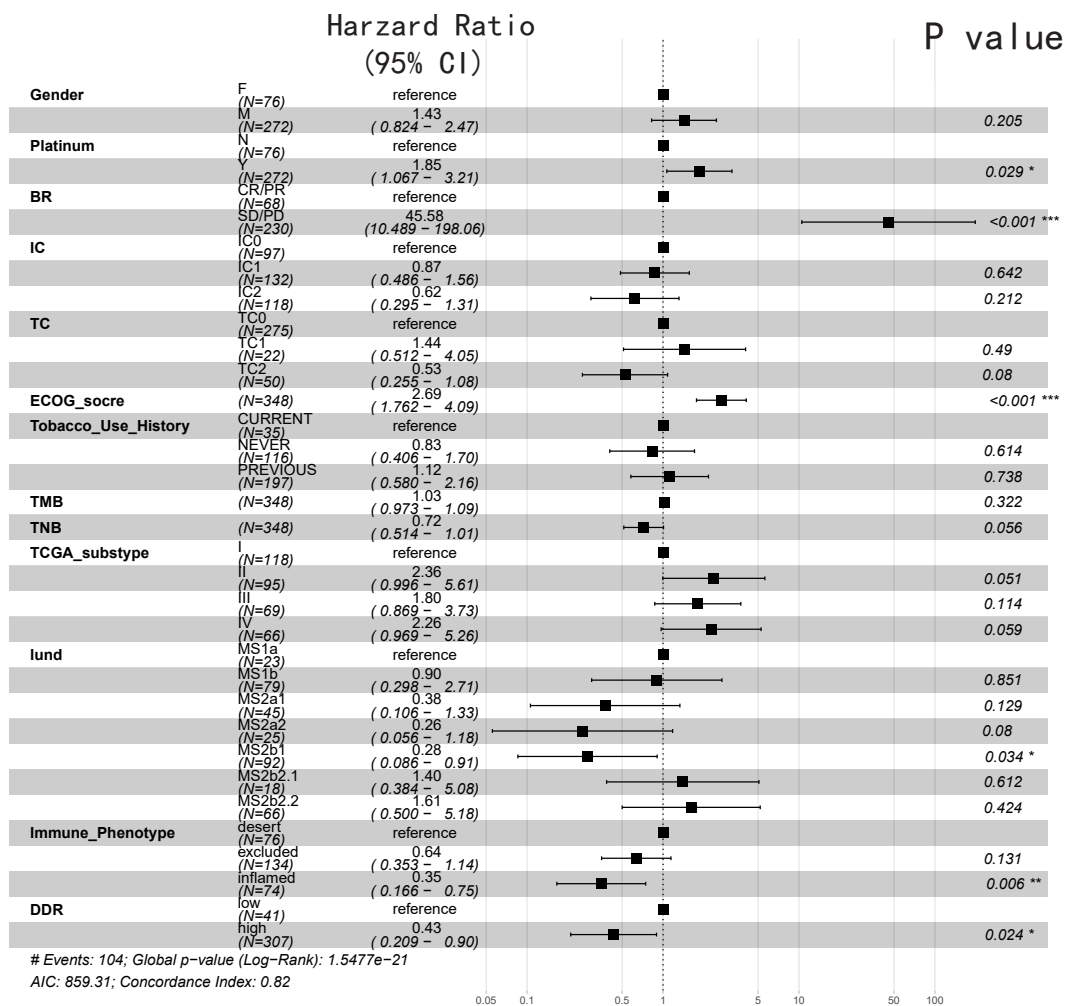

Supplement: Supplementary Figure 3 — Activation of the DDR pathway shows specific prognostic value for ICI-treated mUC. (A) The high activation of eight DDR sub-pathways was accompanied by more responders to ICI. Chi squared test was used for data analysis. (B) Multivariate Cox regression model shows the hazard ratio and 95% confidence interval (95% CI) of the DDR pathway and other clinical parameter or prior reported biomarkers. Higher activation level of the DDR pathway is a protective factor collaborated with the inflamed immune phenotype (DDR: p = 0.024; Immune phenotype, p = 0.006). *p < 0.05; **p < 0.01; ***p < 0.001. [file Image_3.pdf]

A

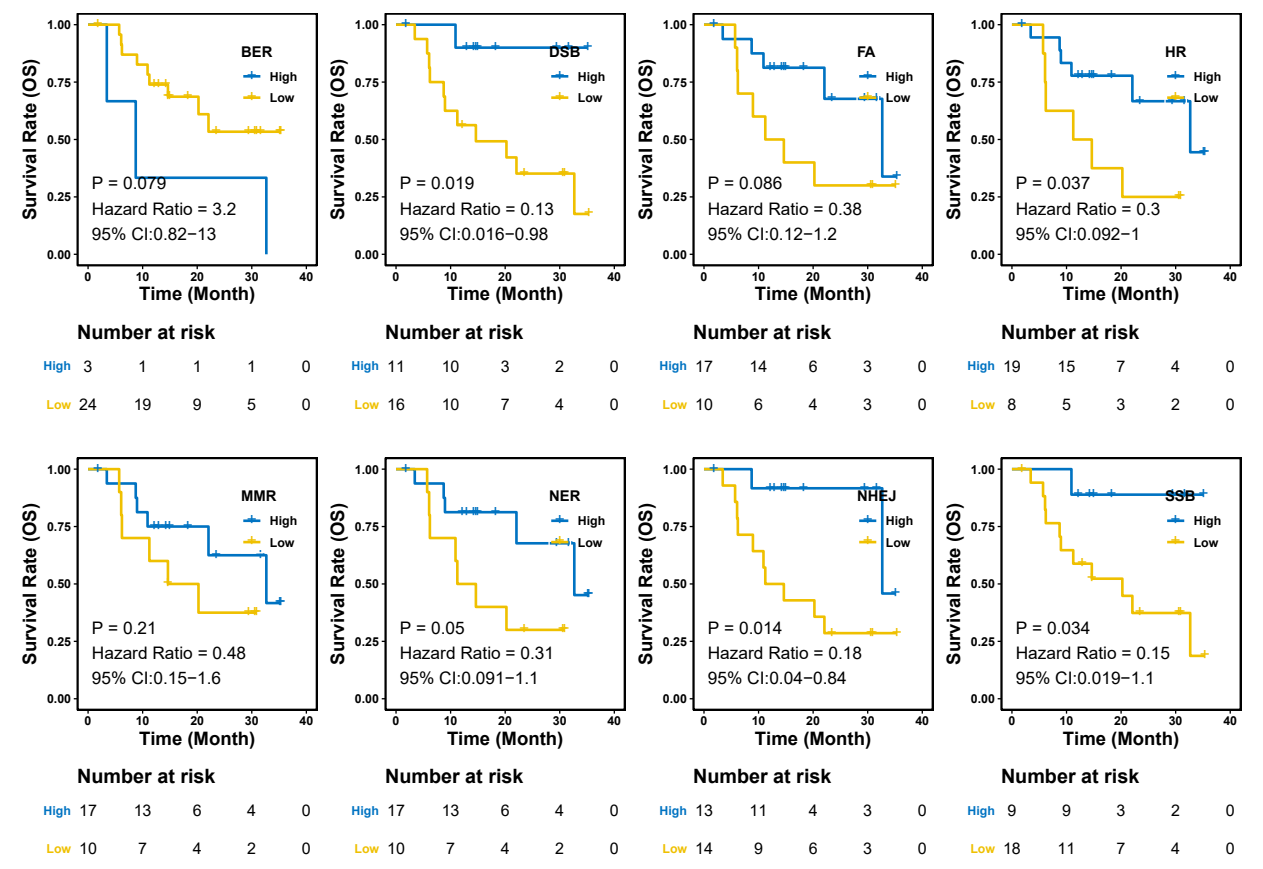

B

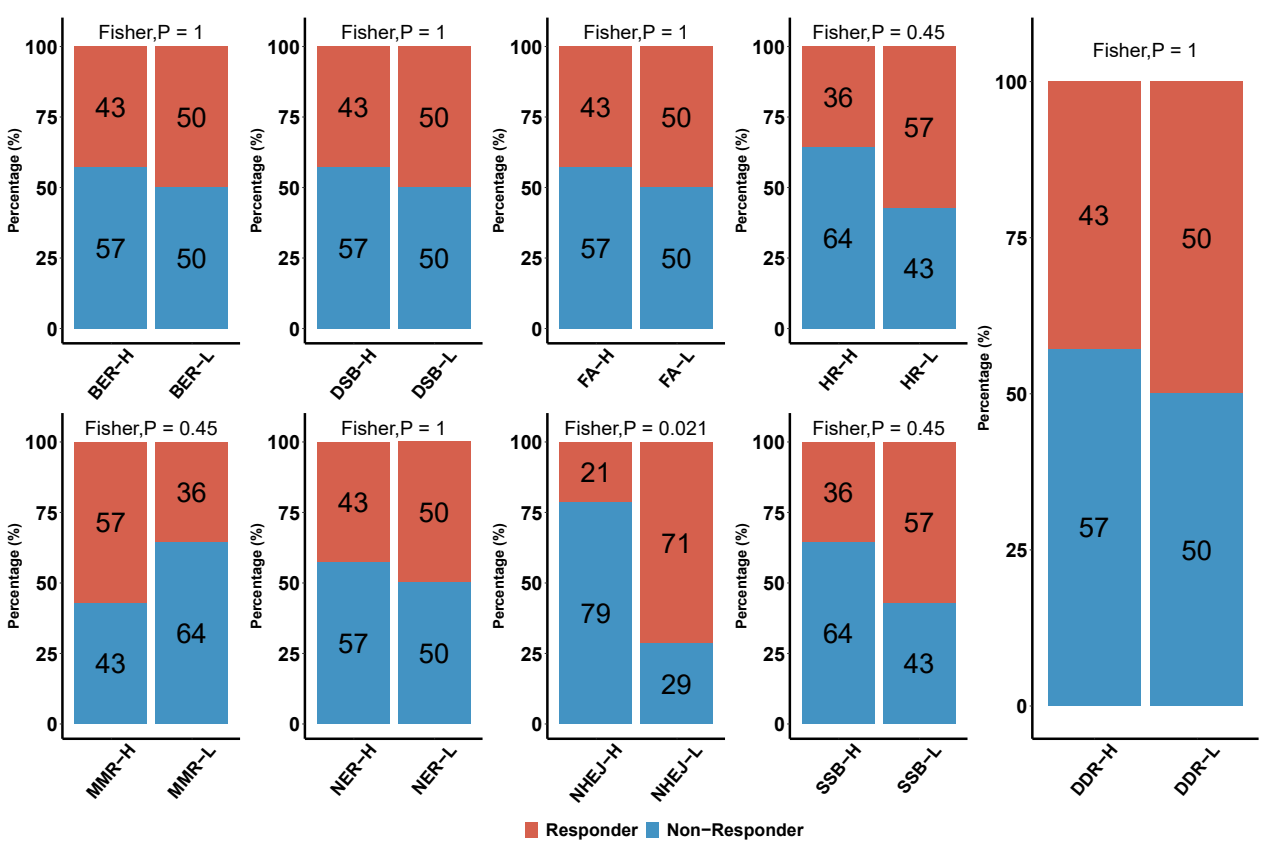

Supplement: Supplementary Figure 4 — Activation of the DDR pathway indicates a favorable prognosis in patients with metastatic melanoma treated with ICIs. (A) KM survival analysis was used to assess the relationship between the activation status of DDR-related pathways and the OS time of patients with metastatic melanoma treated with ICIs. (B) The ratio of responders in groups with high or low activation levels of DDR-related pathways. Fisher’s exact test was used for data analysis. [file Image_4.pdf]

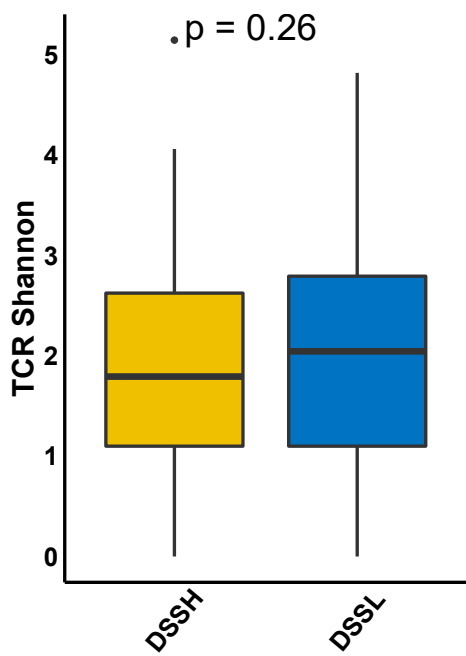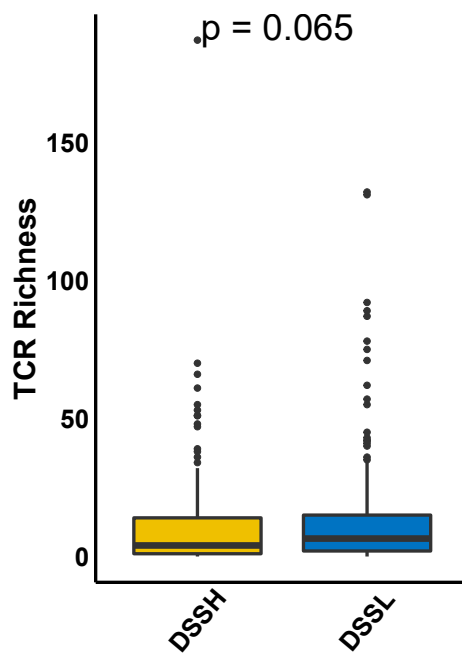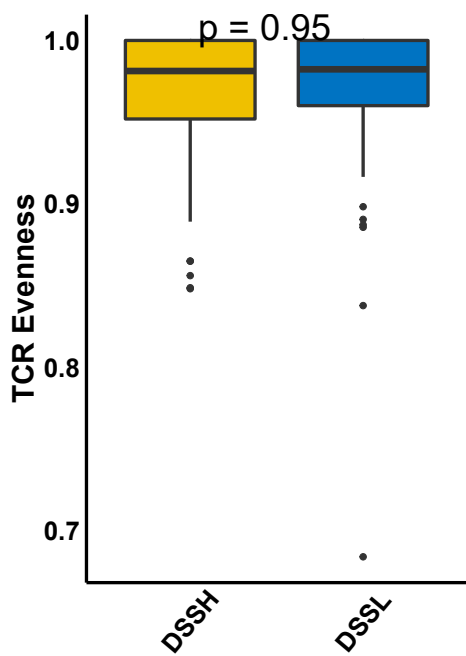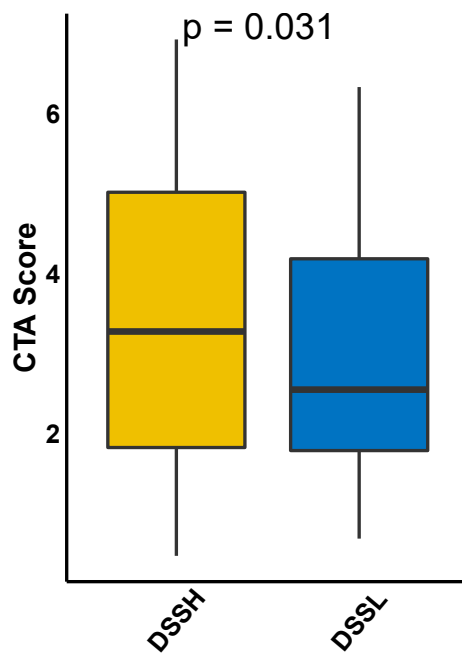

Supplement: Supplementary Figure 5 — Differential analysis between the DSSH and DSSL groups of the TCGA-BLCA cohort in TCR repertoire and cancer testis antigen overall expression (CTA). [file Image_5.pdf]

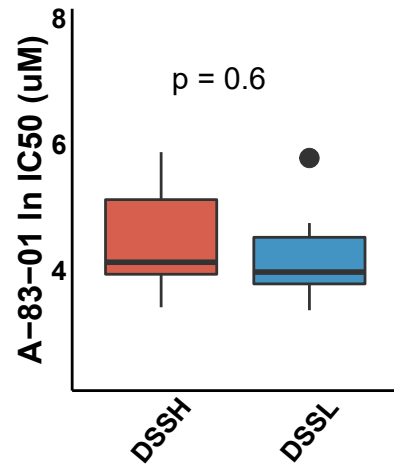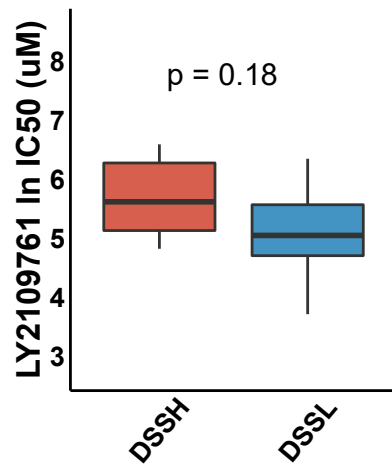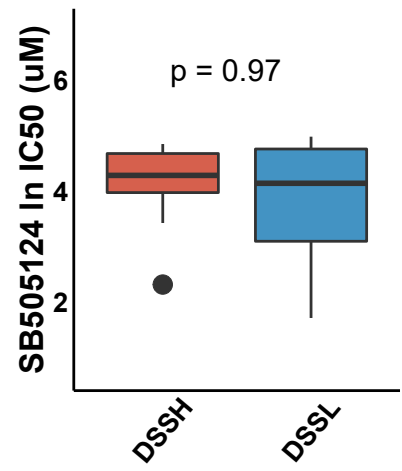

Supplement: Supplementary Figure 6 — Drug sensitivity to three inhibitors of the TGFβ signaling pathway in BC cell lines was not affected by the activation of the DDR pathway. The target of A-83-01 and LY2109761 is TGFB1. The targets of SB505124 are TGFBR1, ACVR1B and ACVR1C. [file Image_6.pdf]
