# Supplementary material for: Activation of the DDR Pathway Leads to the Down-Regulation of the TGFβ Pathway and a Better Response to ICIs in Patients With Metastatic Urothelial Carcinoma
Source: Front Immunol. 2021 Jun 18;12:634741. doi: 10.3389/fimmu.2021.634741 (PMC8253049; doi:10.3389/fimmu.2021.634741)
Supplement: Supplementary Table 1 — List of genes included in DNA damage response (DDR) gene set used for Comparison Analysis (MsigDB). [file Table_1.docx]

Table S1. List of genes included in DNA damage response (DDR) gene set used for Comparison Analysis （MsiDB)

| BER(R-HSA-73884_REACTO  ME_Base_Excision_Repair) | CCNO, POLD3, FEN1, SMUG1, APEX1, LIG1, LIG3, MPG, MUTYH, NTHL1, OGG1, PCNA, POLB, POLD1, POLD2, POLD4,  TDG, XRCC1, MBD4 |
| --- | --- |
| DSB(R-HSA-5696398_REACT  OME_Double_Strand_Break_R epair) | RAD50, XRCC6, H2AFX, LOC389901, LIG1, LIG4, MRE11A, NBN, ATM, TDP1, PRKDC, RAD51, RAD52, RPA1, RPA2, RPA3, LOC651610, BRCA1, BRCA2, TP53BP1, XRCC4, XRCC5,  BRIP1, MDC1 |
| FA(R-HSA-6783310_REACTO  ME_Fanconi_Anemia_Pathwa y) | FANCA, FANCC, FANCD2, FANCE, FANCB, FANCF, FANCG, ZBTB32, UBE2T, ATM, ATR, FANCL, FANCM, RPS27A, LOC648152, LOC651610, LOC651921, BRCA1, BRCA2, RPS27AP11, UBA52, RPS27AP11, USP1, PALB2, C17orf70,  C19orf40 |
| HR(hsa03440_KEGG_Homolo gous_Recombination) | RAD50, H2AFX, LIG1, MRE11A, NBN, ATM, RAD51, RAD52, RPA1, RPA2, RPA3, LOC651610, BRCA1, BRCA2, TP53BP1, BRIP1, MDC1, RAD50, POLD3, EME1, RAD54B, RPA4, MRE11A, NBN, POLD1, POLD2, POLD4, RAD51, RAD51C, RAD51B, RAD51D, RAD52, RPA1, RPA2, RPA3, BLM, SSBP1, BRCA2, TOP3A, XRCC2, XRCC3, SHFM1, MUS81, RAD54L,  TOP3B |
| MMR(hsa03430_KEGG_Mism atch_Repair) | POLD3, MLH3, MSH6, RPA4, LIG1, MLH1, MSH2, MSH3, PCNA, PMS2, POLD1, POLD2, POLD4, RFC1, RFC2, RFC3,  RFC4, RFC5, RPA1, RPA2, RPA3, SSBP1, EXO1 |
| NER(R-HSA-5696398_REACT  OME_Nucleotide_Excision_Re pair) | CDK7, POLD3, ERCC8, DDB1, DDB2, ERCC1, ERCC2, ERCC3, ERCC4, ERCC5, ERCC6, GTF2H1, GTF2H2, GTF2H3, GTF2H4, LIG1, MNAT1, PCNA, POLD1, POLD2, POLE, POLE2, POLR2A, POLR2B, POLR2C, POLR2D, POLR2E, POLR2F, POLR2G, POLR2H, POLR2I, POLR2J, POLR2K, POLR2L, XAB2, POLD4, RAD23B, RFC2, RFC3, RFC4, RFC5, RPA1, RPA2, RPA3,  LOC652672, LOC652857, GTF2H2B, TCEA1, XPA, XPC, CCNH |
| NHEJ(hsa03450_KEGG_Non_  Homologous_End_Joining) | RAD50, DNTT, FEN1, XRCC6, POLL, POLM, LIG4, MRE11A,  PRKDC, DCLRE1C, LOC731751, XRCC4, XRCC5, NHEJ1 |
| SSB(GO:0003697_Single_Stra nded_DNA_Binding) | ERCC1, ERCC4, ERCC5, FUBP1, HMGB2, HNRNPA1, HNRNPA2B1, HNRPDL, IGHMBP2, MLH1, MSH2, MSH3, MYT2, PCBP1, PMS2, POT1, PURA, PURB, RAD23A, RAD23B, RAD51, RAD51AP1, RBMS1, RPA1, RPA2, RPA3, RPA4, SUB1,  TERF2, TERF2IP, TP53, TREX1, WBP11, XPC, YBX1 |
| DDR(merged) | CCNO, POLD3, FEN1, SMUG1, APEX1, LIG1, LIG3, MPG, MUTYH, NTHL1, OGG1, PCNA, POLB, POLD1, POLD2, POLD4, TDG, XRCC1, MBD4, TCEA1, ERCC8, LOC652857, RPA3, RPA3, DDB2, POLR2E, POLR2H, RFC2, XPA, RFC3, ERCC1,  RPA2, RPA2, CCNH, XAB2, ERCC6, POLE, ERCC2, RAD23B, |

POLR2J, POLR2G, DDB1, ERCC5, RPA1, RPA1, LOC652672, GTF2H2, POLR2B, ERCC4, POLR2L, POLR2A, RFC4, GTF2H1, ERCC3, RFC5, GTF2H4, GTF2H2B, POLR2D, POLR2I, CDK7, GTF2H3, POLE2, XPC, POLR2C, MNAT1, POLR2K, POLR2F, MSH3, MSH2, SSBP1, RFC1, EXO1, MLH1, RPA4, PMS2, MSH6, MLH3, PCBP1, PURB, ERCC1, HMGB2, IGHMBP2, RBMS1, RAD23B, HNRNPA2B1, TP53, ERCC5, TERF2IP, PURA, TREX1, ERCC4, WBP11, POT1, RAD51AP1, MYT2, RAD23A, HNRPDL, XPC, FUBP1, RAD51, RAD51, SUB1, HNRNPA1, YBX1, TERF2, RAD50, RAD50, H2AFX, LIG4, PRKDC, XRCC4, LOC651610, NBN, NBN, XRCC6, BRIP1, RAD52, RAD52, TP53BP1, BRCA1, MRE11A, MRE11A, MDC1, TDP1, LIG1, BRCA2, BRCA2, LOC389901, ATM, XRCC5, RAD54L, SHFM1, POLD3, TOP3A, XRCC3, POLD4, RAD51B, SSBP1, TOP3B, RAD51D, MUS81, POLD1, EME1, RPA4, RAD54B, RAD51C, XRCC2, BLM, POLD2, LIG4, PRKDC, NHEJ1, XRCC4, FEN1, POLM, XRCC6, DNTT, LOC731751, POLL, DCLRE1C, XRCC5, FANCA, FANCC, FANCD2, FANCE, FANCB, FANCF, FANCG, ZBTB32, UBE2T, ATM, ATR, FANCL, FANCM, RPS27A, LOC648152, LOC651610, LOC651921, BRCA1, BRCA2, RPS27AP11, UBA52, RPS27AP11, USP1, PALB2, C17orf70, C19orf40
